# Diagnostic Thoracoscopy

**DOI:** 10.1155/DTE.1.195

**Published:** 1995

**Authors:** Julius P. Janssen, Pieter E. Postmus, Johan C. Van Mourik, Miguel A. Cuesta

**Affiliations:** 1 Department of Pulmonary Medicine Canisius Wilhelmina Ziekenhuis Postbus 9015 Nijmegen 6500 GS The Netherlands; 2 Department of Pulmonary Medicine Free University Hospital De Boelelaan 1117 Amsterdam 1081 HV The Netherlands; 3 Department of Surgery Free University Hospital De Boelelaan 1117 Amsterdam 1081 HV The Netherlands

## Abstract

Since the introduction of video-imaging and endoscopic surgical interventions, there is a worldwide renewed interest in thoracoscopy. However, thoracoscopy for diagnosis of pleural and pulmonary disease has been performed for more than 30 years. An overview is presented here of the results and experiences in the past 3 decades of thoracoscopy for diagnosis of pulmonary and pleural disease. Thoracoscopy is a simple and safe method to obtain a diagnosis in case of pleural effusion, pleural mass, or interstitial lung disease. In most cases, it can be performed under local anaesthesia.


*Diagnostic and Therapeutic Endoscopy*, 1995, Vol. 1, pp. 195–200
Reprints available directly from the publisher
Photocopying permitted by license only

© 1995 Harwood Academic Publishers GmbH
Printed in Singapore

### Diagnostic Thoracoscopy

JULIUS P. JANSSEN,* PIETER E. POSTMUS,† JOHAN C. VAN MOURIK,‡ and MIGUEL A. CUESTA‡

**Department of Pulmonary Medicine, Canisius Wilhelmina Ziekenhuis, Postbus 9015, 6500 GS Nijmegen; †Departments of Pulmonary Medicine and ‡Surgery, Free University Hospital, De Boelelaan 1117, 1081 HV Amsterdam, The Netherlands*

*(Received August 15, 1994; in final form November 9, 1994)*

Since the introduction of video-imaging and endoscopic surgical interventions, there is a worldwide renewed interest in thoracoscopy. However, thoracoscopy for diagnosis of pleural and pulmonary disease has been performed for more than 30 years. An overview is presented here of the results and experiences in the past 3 decades of thoracoscopy for diagnosis of pulmonary and pleural disease. Thoracoscopy is a simple and safe method to obtain a diagnosis in case of pleural effusion, pleural mass, or interstitial lung disease. In most cases, it can be performed under local anaesthesia.

**KEY WORDS:** thoracoscopy, pleural disease, pleural effusion, pneumothorax, mesothelioma, tuberculosis, interstitial lung disease

### INTRODUCTION

In 1910 Jacobeus described thoracoscopy as a new method for the investigation of pleural fluid of unknown cause (1). He insufflated air into the pleural cavity after draining the pleural fluid and inspected the pleural surface. Until the 1950s, thoracoscopy was almost only used for adhesiolysis in patients treated with a "therapeutic" pneumothorax for tuberculosis. Early in the 1970s, there was a renewed interest for thoracoscopy in The Netherlands, France, and Germany (2,3). Thoracoscopy was hardly popular in the United States and the United Kingdom until the recent development of disposable equipment for thoracoscopic intervention and video imaging.

Initially, thoracoscopy was used in patients with pleural fluid of unknown origin and pneumothorax (2). Later, several investigators used it for pulmonary biopsy (4–7), and for the diagnosis and treatment of malignant pleural effusions (7,8).

The introduction of video-endoscopic surgery of the abdomen stimulated the development of videothoracoscopy. There is increasing experience in thoracoscopic interventions like thoracoscopic bullectomy, sympathectomy, pericardial fenestration, and (partial) pulmonary resection (9–12). Here, the diagnostic value of thoracoscopy for pleural, pulmonary, and mediastinal diseases is discussed.

### TECHNIQUE

In most patients diagnostic thoracoscopy can be performed either under local or general anesthesia. Severe cardiac and pulmonary disease are relative contraindications (see "Safety and Acceptability"). In the presence of extensive adhesions between the visceral and parietal pleura, it may be impossible to create a pneumothorax and inspect the parietal and visceral pleura. In these cases, a direct or extended thoracoscopy can be performed (13–15). However, these special techniques should only be performed in experienced hands.

For an extensive description of the technique refer specialized literature (4). In short, air is insufflated into the pleural cavity until a nearly complete collapse of the lung is achieved to create sufficient space for a safe introduction of the trocar in the 4th, 5th, or 6th intercostal space in the midaxillary line. Through this trocar an optical telescope is introduced. Depending on the extent of the investigation, a second or even third trocar can be introduced to facilitate the introduction of more instruments. For diagnostic purposes, it is usually sufficient to use local anesthesia with mild sedation and analgesia. For an extensive endoscopic intervention general anesthesia is preferable, using a double lumen tube intubation to achieve a complete collapse of the lung.

Address for correspondence: J.P. Janssen, Department of Pulmonary Medicine, Canisius Wilhelmina Ziekenhuis, Postbus 9015, 6500 GS Nijmegen, The Netherlands

A complete thoracoscopy set consists of a water-column to induce pneumothorax, some trocars with an obturator, an optical telescope, a forceps, and a pair of scissors. Almost exclusively, rigid instruments are used. Some have used flexible bronchoscopes in the past for thoracoscopy (16–18). This technique has not obtained widespread popularity, mainly because it is very difficult to keep an adequate orientation inside the thorax when using a flexible thoracoscope. Recently, a thoracoscope with a rigid shaft and a flexible tip has been developed. This device has not yet been proved to be superior to the rigid equipment (personal experience).

### PNEUMOTHORAX

The incidence of spontaneous pneumothorax (SP) is between 5 and 15 per 100.000 and affects men 4 to 6 times more often than women (19). There is no consensus on the treatment of spontaneous pneumothorax. In many centers pneumothorax first is treated with bedrest with or without chest tube drainage. This gives in a relapse rate of up to 60% (20–23). After the first or second recurrence, the risk of a subsequent recurrence increases considerably to 38% to 83% (20). Even after pleurodesis with tetracycline, which was especially popular in the United States, a recurrence rate of up to 45% has been reported (21,22). The high recurrence rate after conservative treatment for spontaneous pneumothorax has prompted some institutes to perform thoracoscopy in patients with first and recurrent pneumothorax, to select patients that would benefit from surgical resection of bullae (2,24–27). In general, patients with bullae larger than 2 cm are considered good candidates for surgical bullectomy. In case of bullae smaller than 2 cm, only pleurodesis is performed in most cases. During thoracoscopy, bullae may be missed (25,27), which is probably caused by the presence of the majority of bullae at the apex of the lung, which is the hardest part to inspect with the thoracoscope. The most important reason for thoracoscopy in SP is to give information about its probable cause, which may help in the choice of proper treatment for the patient. Although the causal relation between the finding of blebs or bullae and SP has not been sufficiently proved, many assume that their removal is a rational part in the treatment of pneumothorax and helps to prevent recurrence. Only one study showed the relation between occurrence, of blebs and bullae and recurrence of pneumothorax (28). Our data of 85 patients, however, indicate that the occurrence of recurrent SP and the presence of bullae were not correlated, suggesting a lesser impact of the presence of bullae on the clinical course of SP (29).

Recent development of endoscopic stapler devices and the introduction of videothoracoscopy have made it possible to perform interventional procedures under thoracoscopic guidance that traditionally were reserved for thoracotomy. In our institute, we developed a protocol to incorporate surgical procedures in traditional thoracoscopy in SP. In 44 cases of SP, blebs or bullae were found in 66% of patients. Depending on the size of these lesions, treatment consisted of talc pleurodesis and coagulation of lesions smaller than 2 cm, and thoracoscopic bullectomy for lesions larger than 2 cm (30). With the use of videothoracoscopy in SP, diagnosis and treatment can be performed in a single procedure. Thus, in the near future, the need for thoracotomy in patients with SP is expected to be considerably reduced.

### PLEURAL EFFUSION

Analysis of aspirated pleural effusion is the most important investigation in patients with pleural effusion. In a prospective study in 129 patients, a definite diagnosis was obtained in 18% and a probable diagnosis in 56% (31). If repeated aspiration fails to give a diagnosis on a persisting exsudate, a more invasive diagnostic procedure is required. The blind needle biopsy according to Cope or Abrams was the method of choice for more than 30 years, and it still is in some countries. The diagnostic yield of the blind needle biopsy is highly variable. Chrétien found a diagnostic yield of 77% in 213 patients with tuberculosis, being 77% in metastatic carcinoma (n = 178) and only 10% in mesothelioma (32). Others found a lower diagnostic yield of up to 50% for metastatic carcinoma of the pleura (33). Boutin and other authors proved that the diagnostic yield of thoracoscopy was much higher (94% to 96%) in all forms of pleural disease (2,33–37). Thoracoscopy provides the advantage of seeing the extent and the nature of the pleural abnormalities, and biopsies can be taken from macroscopically abnormal as well as from normal pleura. This is an important advantage over the blind needle biopsy. In addition, presence of pleural fluid is necessary for safe use of the blind biopsy needle, whereas this is not the case for thoracoscopy. True-cut biopsies of the pleura guided by CT or ultrasound may serve as an alternative, but the amount of tissue for histological investigation in true-cut biopsy is limited, which makes specific diagnosis in benign disease sometimes difficult (38).

### MALIGNANT PLEURAL EFFUSION

The diagnostic yield of thoracocenthesis and blind pleural biopsy in malignant pleural disease correlates strongly to the stage of disease (34). In many studies, the superiority of thoracoscopy in the diagnosis of malignant pleural disease over thoracocentesis or blind pleural biopsy has been shown (34–35,39).

In a large study concerning 1000 patients with pleural effusion, no diagnosis was obtained after repeated thoracocenthesis and blind pleural biopsy in 215 patients (22%) (34). One hundred fifty patients appeared to have malignant pleural disease of whom 131 (87%) were diagnosed by thoracoscopic pleural biopsy. After improvement of his equipment, the diagnostic yield in Boutin's study raised to 97%. Such a high diagnostic yield of thoracoscopy in pleural effusion also was found in the studies of Canto (94%), Hacker (80%), Menzies (96%), Ohri (86%), Oldenburg (86%), Page (98.9%), Swierenga (95%), and Weissberg (94%) (2,35–37, 40–43).

In a review of 4301 reported cases of diagnostic thoracoscopy for chronic pleural effusion in 21 studies, a correct pathologic diagnosis was obtained in 1331 (92.5%) of 1472 cancer cases (4). The diagnostic yield of thoracoscopy in pleural malignancy is much higher than the combination of blind pleural biopsy and thoracocenthesis for several reasons:

1. Thoracoscopy allows inspection of the complete parietal pleura and three quarters of the visceral pleura.
2. Hence, biopsies can be taken under visual control at sites where tumor appears to be localized.
3. Multiple biopsies can be taken, which are of greater size and depth than blind biopsies.
4. In a study that assessed the location of pleural malignancy, Canto found the costal pleura, which is the only part that can be reached by the blind biopsy needle, to be involved in only 53% of cases (44). In an autopsy study of 53 patients with metastatic pleural malignancy, Meyer found involvement of only the visceral pleura in 17 (32%) cases (45).

### BRONCHIAL CARCINOMA

Pleural effusion in bronchial carcinoma deserves special attention, because metastatic spread to the pleura makes thoracotomy and pulmonary resection useless. However, not all pleural effusions in these patients are caused by pleural metastasis; also, the effusion can be parapneumonic or secondary to venous or lymphatic obstruction. To assess pleural metastatic disease and consequently prevent unnecessary thoracotomy thoracoscopy is the procedure of choice in these patients. In a compilation of two series of 99 patients with bronchial carcinoma and pleural effusion, no thoracoscopic evidence of pleural metastasis was found in 13 (13%) (46,47). Normal cytology after thoracocenthesis in these patients does not rule out pleural metastasis.

Beside pleural effusion in bronchial cancer, thoracoscopy can provide information about tumor invasion into the mediastinum or the thoracic wall. In cases of extended invasion of the mediastinum diagnosed by thoracoscopy, unnecessary thoracotomy can be avoided. The use of thoracoscopy in the assessment of lymph node metastasis is discussed in another chapter (see "Mediastinal Masses").

In our institute, in the last year thoracoscopy was performed for staging of lung cancer in 10 patients; in 5 cases for assessment of N-status and in 5 cases for assessment of T-status. Irresectability of bronchial cancer was demonstrated in 6 (60%) of 10 patients.

### MALIGNANT MESOTHELIOMA

The local incidence of malignant mesothelioma is highly variable and depends on previous exposure to asbestos (48). Similar to other malignant pleural diseases, the diagnostic yield of thoracoscopy is superior to the combination of blind pleural biopsy and thoracocenthesis (48). It is important to provide many representative biopsy samples to the pathologist, because the histologic diagnosis of mesothelioma often is difficult (4,48). Thoracoscopy allows for some prediction of survival, because involvement of the visceral pleura is associated with a very unfavourable prognosis (48,49).

### TUBERCULOUS PLEURAL EFFUSIONS

Loddenkemper performed a prospective study in 100 patients of whom the diagnosis of TB could be immediately established on histologic examination of thoracoscopic biopsies in 94%, compared with 38% of blind needle biopsies (50). In a large review of the literature on 1325 cases, Loddenkemper found an average diagnostic yield of blind needle biopsy in TB of 69%, with a range of 28% to 88% (51). Because of the high diagnostic yield on thoracoscopic histologic biopsies, unnecessary delay of antituberculous therapy is minimized. Thoracoscopy in the early course of disease may demonstrate the specific pattern of granulomas, which may disappear spontaneously after 3 to 4 weeks (4).

### INTERSTITIAL LUNG DISEASE

The differential diagnosis of interstitial lung disease is extensive, and it is often difficult to establish a definitive diagnosis. Immunological evaluation and bronchoalveolar lavage are important diagnostic tools in these patients. Usually, tissue for histological investigation is obtained by multiple transbronchial biopsies through the fiberoptic bronchoscope. The diagnostic yield of this procedure is variable and depends on the underlying disease (52,53). In a group of 53 patients, Wall and coworkers found transbronchial biopsy to be diagnostic in only 20 (37%) cases. Sarcoidosis can be diagnosed in 97% of the cases if four to six biopsies are performed (54,55). The procedure is much less accurate in categorizing nonspecific pneumonitis or fibrotic processes (53). If transbronchial biopsy does not provide a clear diagnosis, one should consider open lung biopsy by thoracotomy. An alternative is thoracoscopic lung biopsy, either obtained by biopsy forceps or wedge resection. The advantages of this technique over thoracotomy are obvious: less morbidity and shorter hospitalization. Complications such as air embolus, bleeding, or persistent pneumothorax are rare (5–7).

Results of two large studies on thoracoscopic lung biopsy were published in 1982 (6,7). Dijkman and coworkers performed thoracoscopic lung biopsies in 63 patients; a histologic diagnosis was obtained in 57 (90%) (6). In a series of 20 patients with diffuse pulmonary disease, Boutin found a diagnostic yield of 100% (7). In his study, one to eight biopsies from different parts of the lung were taken.

Recently, two studies reported of thoracoscopic lung biopsy obtained by wedge resection technique using the endoscopic GIA stapler (56,57). The diagnostic yield in these studies was 100% and 95%. Although biopsy specimens obtained by forceps technique are smaller than those obtained by wedge resection, the diagnostic yield does not differ significantly.

Recently we have begun to use the 5-mm coagulating biopsy forceps developed by Boutin (Wolf Company, Knittlingen, Germany) for pulmonary biopsies in interstitial lung disease. With the technique of simultaneous cutting and coagulating originally described by Boutin, there was no air leakage or bleeding at the biopsy site in our experience (4). After this procedure, the histopathological features of the biopsy specimen are well conserved, and in our experience sufficient lung tissue can be provided to the pathologist for a correct histopathological diagnosis.

This forceps biopsy technique is especially useful in children. In very young patients, the thoracoscopic stapler device cannot be used, because the intercostal space does not allow introduction of a 12-mm trocar. The 5-mm biopsy forceps fits in a 7-mm trocar, which can be easily introduced into the intercostal space of a child (personal experience in two children, 7 and 10 years old).

### DIAGNOSTICS OF MEDIASTINAL MASSES

Histologic diagnosis of a mediastinal mass is not easy to obtain without an invasive surgical procedure. Fine needle aspiration provides an accurate diagnosis in only a minority of cases (58). In all other cases, diagnostic sternotomy or thoracotomy used to be the procedure of choice. Kern and coworkers recently performed videothoracoscopy in 22 patients with mediastinal masses (59). In 19 (86%) of these an accurate tissue diagnosis could be obtained without need for an open procedure.

A special case of mediastinal mass is an enlarged lymph node in a patient with lung cancer. Usually, cervical mediastinoscopy is performed to sample lymph node tissue for diagnosis. However, lymph nodes in the aortic window are unaccessible by cervical mediastinoscopy.

Traditionally, anterior mediastinotomy or parasternal mediastinoscopy is performed in these cases, but left-sided thoracoscopy may be a suitable alternative as has recently been shown by Landreneau and in our own experience (60,61). To facilitate the thoracoscopic inspection of the aortic window, we used an ultrasonographic transducer, specially developed for endoscopic use (diameter, 10 mm; frequency, 7.5 Mhz; Aloka Company, Japan). Thoracoscopic ultrasonography of the aortic window served to localize enlarged lymph nodes, defined their relation to vascular structures, and permitted a guided biopsy (61).

### SAFETY AND ACCEPTABILITY OF DIAGNOSTIC THORACOSCOPY

Complications of thoracoscopy are very infrequent in experienced hands. An air embolus may occur when during the insufflation of air for creating a pneumothorax the needle is not properly positioned and air is insufflated into a blood vessel. Severe hemorrhage after a biopsy of the lung is very uncommon, the blood vessels in the peripheral parts of the lung are small, and the elastic properties of the lung result in contraction of the vessels after a biopsy has been taken. Only in case of a biopsy of the parietal pleura is there risk of a major bleeding if an intercostal artery is damaged. Small bleeding is easily managed by electrocoagulation or Nd-YAG laser. Persistent air leakage is a known complication of a biopsy of a stiff lung in case of lung fibrosis or emphysema (4).

Diagnostic thoracoscopy can be safely performed under local anaesthesia, as has been reported by several authors (35,37,42,62). Local anesthesia is well tolerated by the patient. The most frequent problem is a sharp transient pain during parietal pleural biopsy, which can be relieved by pretreatment with morphine, and local application of lidocaine before biopsy (42).

For a safe thoracoscopy procedure under local anesthesia, several precautions have to be taken. Pulse and 02 saturation (oximeter) should be monitored. Supplementary oxygen should be available, as well as an intravenous route for immediate resuscitative measures in case of cardiac problems. To manage complications of bleeding, electrocoagulation should be immediately at hand.

Several studies showed that thoracoscopy can be safely performed under local anaesthesia. In four studies concerning 819 patients, no deaths were reported (16,37,63,64).

Complications were subcutaneous emphysema (39), transient cardiovascular complications (10), empyema (2), fever (2), excessive bleeding (1), and air embolism (1).

Ohri reviewed extensively the complications after thoracoscopy under general anaesthesia (41). In his own series of 100 patients, death occurred in 5% (5 patients; cause of death was cardiac arrest in 2, malignant cachexia in 2, and respiratory failure in 1). In a compilation of five series concerning 549 patients who underwent diagnostic thoracoscopy under general anesthesia, there were 13 (2.3%) deaths.

Causes of death were advanced malignancy in 8, cardiac arrest in 3, pulmonary embolus in 1, and respiratory failure in 1 (34,36,41,65,66).

The thoracoscopic procedure itself may be complicated by the presence of multiple adhesions between visceral and parietal pleura. In these cases, thoracoscopy may be complicated by a diminished view and bleeding in case of rupture of an adhesion. If the view is diminished by adhesions, these can be cut with a coagulating forceps. In some cases, however, it may be impossible to introduce the thoracoscope at all because of extended adhesions. In these cases an, incision of 3 to 4 cm can be made. Afterward, the thoracoscopist introduces his index finger to create a space between the visceral and parietal pleura, separating the two by rotation of the finger. This technique, called extended thoracoscopy, was decribed by us in an earlier publication (13). In a retrospective study of 22 patients with extended or complete adhesion of the visceral and parietal pleura, we found a diagnostic yield of 84% using extended thoracoscopy. No major complication occurred; however, we recommend this technique only to experienced thoracoscopists.

### CONCLUSIONS

Diagnostic thoracoscopy in an important tool in the diagnosis of pleural and pulmonary diseases. It is a safe procedure that is hardly invasive, and it provides the visual information and the diagnostic yield of a thoracotomy. Nowadays, the technical possibilities of diagnostic thoracoscopy are importantly extended by video-imaging and the use of endoscopic stapling devices. Presently, the role of pulmonologists and thoracic surgeons in thoracoscopy is a matter of discussion. Diagnostic thoracoscopy has been developed by European pulmonologists in the past. Concerning its classical indications, thoracoscopy also should be performed by the pulmonologist in the future, because of his historical experience, his outstanding knowledge of pleural and pulmonary diseases, and his familiarity with endoscopy equipment. However, in the rapidly changing field of interventional thoracoscopy, close communication and cooperation with the thoracic surgeon are indispensable.

### REFERENCES

1. Jacobeus HC. Uber die Mögligkeit die Zystoscopie bei Untersuchung seroser Höhlen anzuwenden. Münchener Medizinischer Zschr 1910;40:2090–2092.
2. Swierenga J, Wagenaar JPM, Bergstein PGM. The value of thoracoscopy in the diagnosis and treatment of diseases affecting the pleura and lung. Pneumologie 1974;151:11–18.
3. Brandt HJ. Diagnostik der Pleuraerkrankungen einschliesslich Thorakoskopie und Biopsie. Thoraxchirurgie 1974;22:371–380.
4. Boutin C, Viallat JR, Aelony Y. Practical Thoracoscopy Springer Verlag, 1991.
5. Kapsenberg PD. Thoracoscopic biopsy under visual control. Poumon Coeur 1981;37:313–316.
6. Dijkman JH, Meer van der JWM, Bakker W, Wever AMJ, Broek van der PJ. Transpleural lung biopsy by the thoracoscopic route in patients with diffuse interstitial pulmonary disease. Chest 1982;82:76–83.
7. Boutin C, Viallat JR, Cargnino P, Rey F. Thoracoscopic lung biopsy. Chest 1982;82:44–48.
8. Aelony Y, King R, Boutin C. Thoracoscopic talc poudrage pleurodesis for chronic recurrent pleural effusions. Ann Intern Med 1991;115:778–782.
9. Miller JI. Therapeutic thoracoscopy: new horizons for an established procedure. Ann Thorac Surg 1991;52:1036–1037.
10. Landreneau RJ, Herlan DB, Johnson JA, Boley TM, Nawarawong W, Ferson PF. Thoracoscopic Nd-YAG laser-assisted pulmonary resection. Ann Thorac Surg 1991;52:1176–1178.
11. Wakabayashi A, Brenner M, Wilson AF, Tadir Y, Berns M. Thoracoscopic treatment of spontaneous pneumothorax using carbon dioxide laser. Ann Thorac Surg 1990;50:786–790.
12. Massad M, LoCicero J, Matano J, et al. Endoscopic thoracic sympathectomy: evaluation of pulsatile laser, non-pulsatile laser, and radiofrequency-generated thermocoagulation. Lasers Surg Med 1991;11:18–25.
13. Janssen JP, Boutin C. Extended thoracoscopy: a method to be used in case of pleural adhesions. Eur Resp J 1992;5:763–766.
14. Lewis RJ, Kunderman PJ, Sisler GE, Mackenzie JW. Direct diagnostic thoracoscopy. Ann Thorac Surg 1976;21:536–539.
15. Maassen W. Thoracoscopie et biopsie pulmonaire sans pneumothorax initial. Poumon Coeur 1981;37:317–320.
16. Davidson AC, George RJ, Sheldon CD, Sinha G, Corin B, Geddes DM. Thoracoscopy: assessment of a physician service and comparison of a flexible bronchoscope used as thoracoscope with a rigid thoracoscope. Thorax 1988;43:327–332.
17. Gwin E, Pierce G, Boggan M, et al., Pleuroscopy and pleural biopsy with the flexible fiberoptic bronchoscope. Chest 1975;67:527–31.
18. Williams T, Thomas P. The diagnosis of pleural effusions by fiberoptic bronchoscopy and pleuroscopy. Chest 1981;80:566–569.
19. Neal JF, Vargas G, Smith DE, Bechara FA, Edwards WS. Bilateral bleb excision through median sternotomy. Am J Surg 1979;138:794–797.
20. Getz SB, Beasley WE. Spontaneous pneumothorax. Am J Surg 1983;45:823–827.

21. Almind M, Lange P, Viskum K. Spontaneous pneumothorax: comparison of simple drainage, talc pleurodesis, and tetracycline pleurodesis. Thorax 1989;44:627–630.
22. Guérin JC, Boniface E. Les méthodes de pleurodèse. Revue du Praticien 1990;40:1854–1856.
23. O'Rourke JP, Yee ES. Civilian spontaneous pneumothorax. Chest 1989;96:1302–1306.
24. Vanderschueren RGJRA. Le talcage pleural dans le pneumothorax spontané. Poumon Coeur 1981;37:273-276.
25. Olsen PS, Andersen HO. Long-term results after tetracycline pleurodesis in spontaneous pneumothorax. Ann Thorac Surg 1992;53:1015–1017.
26. Verschoof AC, Ten Velde GPM, Greve LH, Wouters EFM. Thoracoscopic pleurodesis in the management of spontaneous pneumothorax. Respiration 1988;53:197–200.
27. Brekel van de JA, Duurkens VAM, Vanderschueren RGJRA. Pneumothorax: results of thoracoscopy and pleurodesis with talc poudrage and thoracotomy. Chest 1993;103:345–347.
28. Torres Lanzas J, Rivas de Andres JJ. Recidiva del Neumothorax espontaneo y su relacion con la presencia de bullas. Arch Bronconeumol 1985;21:212–216.
29. Janssen JP, Schramel FMNH, Sutedja TG, Cuesta MA, Postmus PE. Videothoracoscopic appearance of first and recurrent pneumothorax. (Submitted).
30. Janssen JP, Van Mourik J, Cuesta MA, Sutedja G, Gigengack K, Postmus PE. Treatment of patients with spontaneous pneumothorax during videothoracoscopy. Eur Respir J 1994;7:1281–1284.
31. Collins TR, Sahn SA. Thoracocenthesis. Chest 1987;91:817–822.
32. Chrétien J, Danel CJ. Needle pleural biopsy. In: Chrétien J, Bignon J, Hirsch A (eds). The pleura in Health and Disease. Marcel Dekker, 1985.
33. Astoul Ph, Boutin C, Seitz B. Diagnostic des pleurésies. Revue du Praticien 1990;40:1829–1836.
34. Boutin C, Viallat JR, Cargnino P, Farisse P. Thoracoscopy in malignant pleural effusions. Am Rev Resp Dis 1981;124:588–592.
35. Canto A, Blasco E, Casillas M. Thoracoscopy in the diagnosis of pleural effusion. Thorax 1977;32:550–554.
36. Page RD, Jeffrey RR, Donnelly RJ. Thoracoscopy: a review of 121 consecutive surgical procedures. Ann Thorac Surg 1989;48:66–68.
37. Menzies R, Charbonneau M. Thoracoscopy for the diagnosis of pleural disease. Ann Intern Med 1991;114:271–276.
38. Ikezoe J, Morimoto S, Arisawa J, Takashima S, Kozuka T, Nakahara K. Percutaneous biopsy of thoracic lesions: value of sonography for needle guidance. Am J Radiol 1990;154:1181–1185.
39. Loddenkemper R, Grosser H, Gabler A, Mai J, Preussler H, Brandt HJ. Prospective evaluation of biopsy methods in the diagnosis of malignant pleural effusions: intrapatient comparison between pleural fluid cytology, blind needle biopsy and thoracoscopy. Am Rev Resp Dis 1983;127 (suppl 4):114.
40. Hucker J, Bhatnagar NK, Al-Jilaihawi AN, Forrester-Wood CP. Thoracoscopy in the diagnosis and management of recurrent pleural effusions. Ann Thorac Surg 1991;52:1145–1147.
41. Ohri SK, Oswal SK, Townsend ER, Fountain SW. Early and late outcome after diagnostic thoracoscopy and talc pleurodesis. Ann Thorac Surg 1992;53:1038–1041.
42. Oldenburg FA, Newhouse MT. Thoracoscopy: a safe accurate diagnostic procedure using the rigid thoracoscope and local anesthesia. Chest 1979;75:45–50.
43. Weissberg D, Kaufman M. Diagnostic and therapeutic thoracoscopy. Chest 1980;78:732–735.
44. Canto A, Rivas J, Saumench J, Morera R, Moya J. Points to consider when choosing a biopsy method in cases of pleurisy of unknown origin. Chest 1983;84:176–179.
45. Meyer PC. Metastatic carcinoma of the pleura. Thorax 1966;21:437.
46. Canto A, Ferrer G, Romagosa V, Moya J, Bernat R. Lung cancer and pleural effusion: clinical significance and study of pleural metastatic locations. Chest 1985;87:649–652.
47. Weissberg D, Kaufmann M, Schwecher I. Pleuroscopy in clinical evaluation and staging of lung cancer. Poumon Coeur 1981;37:241–243.
48. Boutin C, Farisse P, Choux R, Carnigno P, Castaigne JP. Intérêt de la pleuroscopie dans le diagnostic des mésothéliomes malins diffus. Revue Française des maladies respiratoires 1976;4:972–974.
49. Adams VI, Unni KK, Muhm JR, Jett JR, Ilstrup DM, Bernatz PE. Diffuse malignant mesothelioma of the pleura: Diagnosis and survival in 92 cases. Cancer 1986;58:1540–1551.
50. Loddenkemper R, GRsser H, Mai J, Preussler H, Wundschock M, Brandt HJ. Diagnostik des tuberkulösen Pleuraergusses: prospektiver Vergleich laborchemischer, bakteriologischer, zytologischer und histologischer Untersuchungsergebnisse. Prax Klin Pneumol 1983;37:1153–1156.
51. Loddenkemper R. Thoracoscopy: results in non-cancerous and idiopa thic pleural effusions. Poumon Coeur 1981;37:261–264.
52. Wall CP, Gaensler EA, Carrington CB, Hayes JA. Comparison of transbronchial and open biopsies in chronic infiltrative lung diseases. Am Rev Resp Dis 1981;123:280–285.
53. Shure D. Transbronchial biopsy and needle aspiration. Chest 1989;95:1130–1138.
54. Koerner SK, Sakowitz AJ, Appelman RI, Becker NH, Schoenbaum SW. Transbronchial lung biopsy for the diagnosis of sarcoidosis. New Engl J Med 1975;293:268–70.
55. Gilman MJ, Wang KP. Transbronchial lung biopsy in sarcoidosis: an approach to determine the optimal number of biopsies. Am Rev Respir Dis 1980;122:721–724.
56. Ferson PF, Landreneau RJ, Dowling RD, et al. Comparison of open versus thoracoscopic lung biopsy for diffuse infiltrative pulmonary disease. J Thorac Cardiovasc Sur 1993;106:194–9.
57. Bensard DD, McIntyre RC, Waring BJ, Simon JS. Comparison of Videothora coscopic lung biopsy to open lung biopsy in the diagnosis of interstitial lung disease. Chest 1993;103:765–770.
58. Linder J, Olsen GA, Johnston WW. Fine-needle aspiration biopsy of the mediastinum. Am J Med 1986;81:1005–1008.
59. Kern JA, Daniel TM, Tribble CG, Silen ML, Rodgers BM. Thoracoscopic diagnosis and treatment of mediastinal mass. Ann Thorac Surg 1993;56:92–96.
60. Landreneau RJ, Hazelrigg SR, Mack M, et al.: Thoracoscopic mediastinal lymph node sampling: Useful for mediastinal lymph node stations inaccessible by cervical mediastinoscopy. J Thorac Cardiovasc Surg 1993;106:554–558.
61. Janssen JP, Cuesta MA, Tan TP, Postmus PE. Ultrasonographic guided biopsy of subaortic lymph nodes during thoracoscopy. Chest 1994;106:1927–28.
62. Vanderschueren RGJRA. Thoracoscopie sous anesthésie local. Poumon Coeur 1981;37:21–23.
63. Enk B, Viskum K. Diagnostic thoracoscopy. Eur Respir J 1981;62:344–351.
64. DeCamp PT, Mosely PW, Scott ML, Hatch HB Jr. Diagnostic thoracoscopy. Ann Thorac Surg 1973;16:79–84.
65. Daniel TM, Tribble CG, Rodgers BM. Thoracoscopy and talc poudrage for pneumothoraces and effusions. Ann Thorac Surg 1990;50:186–189.
66. Weissberg D, Kaufmann M, Zurkowski Z. Pleuroscopy in patients with pleural effusions and pleural masses. Ann Thorac Surg 1980;29:205–208.

